# A Population Getting Shorter

**Published:** 1843-04

**Authors:** 


We read the following article in the London Lancet, (Nov. 26, 1842):

*A Population getting Shorter.*—"In the department of Finisterre (Brittany), the use of ardent spirits seems to increase, and to be attended with some peculiar effects on the population. In the two arrondissements of Quimper and Quimperlé, the spirituous liquors imported increased from 1,869 hectolitres in 1825, to 3,985 in 1839, and, corresponding with this increase, the average stature of young persons subject to military service is said to have diminished until it had become 22 millimetres (about an inch) less in 1838 than in 1818. A much greater number of individuals was also found unfit for service in the former than in the latter-named year. Gin and camomile have long been in repute in England for stunting the growth of little dogs."

As a part of the general subject of Hygiene, and in connexion with the principles and practice which should govern and prevail in the temperance reform, we have had frequent occasion to correct the common, we might almost designate it vulgar, opinion of the temperance of the French, as regards the use of alcoholic liquors,—because, forsooth, France is a country of vines and of wine manufactory. In reference to the very province noticed in the above article, from the Lancet, we may refer our readers to a volume of ours published last year, "On Regimen and Longevity: Comprising Materia Alimentaria, Dietetic Usages, and the Influence of Civilization on Health and Duration of Life." pp. 42[illegible]. 12mo.

At page 78 of this work we find the following: "It will surprise many of my readers, who cannot connect ideas of intemperance in the use of strong drinks, with the habits of Frenchmen of any class, to be told, on very competent authority (Perrier, of Brest, Preface of Foreign Appendix to Report from Commissioners on the Poor Laws, p. 68), in reference to the people of Brittany: 'The principal cause of misery is inebriety: its frequency among the lower orders keeps them in poverty. The "cabaret" (wine and brandy shops) absorb a great portion of their earnings. This vice is not confined to men; the women partake of it. It has decreased within the last five years, but is still (1834) considerable.' The favorable prospect held out in this last sentence does not, however, seem to have been realised.

"I shall refer again to this dark feature of French dietetic regimen, in connexion with health, when I speak of grapes and their fermented juice or wine, and also in a subsequent chapter upon drinks. It will then be shown, by statistical documents of French preparation, that the people of France, in the use of wine, cider, beer, and brandy, for drink, consume in this way, per individual, more than the people of Great Britain and Ireland, per individual. The evils growing out of the immense indulgence in alcoholic drinks, in the impoverishment, diseases, and demoralization of the French people, will also be exhibited, by reference to the communications and reports of their own physicians, statists, and philanthropists." p. 80.—*Bulletin of Med. Science*, Feb. 1843.

